# Factors correlated with running economy among elite middle‐ and long‐distance runners

**DOI:** 10.14814/phy2.15076

**Published:** 2021-10-25

**Authors:** Cecilie E. Hansen, Martin Stensvig, Jacob Wienecke, Chiara Villa, Jakob Lorentzen, John Rasmussen, Erik B. Simonsen

**Affiliations:** ^1^ Department of Neuroscience University of Copenhagen Copenhagen N Denmark; ^2^ Department of Sport and Nutrition University of Copenhagen Copenhagen N Denmark; ^3^ Department of Forensic Medicine University of Copenhagen Copenhagen Ø Denmark; ^4^ Department of Materials and Production Aalborg University Aalborg Ø Denmark

**Keywords:** Achilles tendon moment arm, biomechanics, fascicle length, running economy, stiffness

## Abstract

Running economy (RE) at a given submaximal running velocity is defined as oxygen consumption per minute per kg body mass. We investigated RE in a group of 12 male elite runners of national class. In addition to RE at 14 and 18 km h^−1^ we measured the maximal oxygen consumption (VO_2max_) and anthropometric measures including the moment arm of the Achilles tendon (*L*_Ach_), shank and foot volumes, and muscular fascicle lengths. A 3‐D biomechanical movement analysis of treadmill running was also conducted. RE was on average 47.8 and 62.3 ml O_2_ min^−1^ kg^−1^ at 14 and 18 km h^−1^. Maximal difference between the individual athletes was 21% at 18 km h^−1^. Mechanical work rate was significantly correlated with VO_2_ measured in L min^−1^ at both running velocities. However, RE and relative work rate were not significantly correlated. *L*_Ach_ was significantly correlated with RE at 18 km h^−1^ implying that a short moment arm is advantageous regarding RE. Neither foot volume nor shank volume were significantly correlated to RE. Relative muscle fascicle length of m. soleus was significantly correlated with RE at 18 km h^−1^. Whole body stiffness and leg stiffness were significantly correlated with *L*_Ach_ indicating that a short moment arm coincided with high stiffness. It is concluded that a short *L*_Ach_ is correlated with RE. Probably, a short *L*_Ach_ allows for storage of a larger amount of elastic energy in the tendon and influences the force–velocity relation toward a lower contraction velocity.

## Graphical abstract

Running economy was found to be significantly related to Achilles tendon moment arm, foot length ratio, whole body stiffness and leg stiffness, and muscular fascicle length.

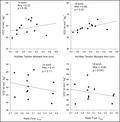

RUNNING ECONOMY (RE) at a specific submaximal running velocity is defined as oxygen consumption (VO_2_) per minute per kg body mass. RE can also be normalized with respect to distance as VO_2_ kg^−1^ km^−1^. Normalization to body mass allows for comparisons between individuals. RE is a complex measure, which reflects the combined functioning of biomechanical, anatomical, metabolic and cardio‐respiratory factors (Tawa & Louw, 2018). Even among well‐trained runners, RE can be seen to differ up to approximately 30% between individuals (Barnes et al., 2014; Larsen, 2003; Saunders et al., 2004a; Scholz et al., 2008). This makes RE a most decisive performance factor in competitive middle‐ and long‐distance running together with VO_2max_ and “Utilization of VO_2max_,” which often refers to the relative load corresponding to “onset of blood lactate.” (Larsen & Sheel, 2015).

A few anatomical measures have been shown to relate to RE. One is the moment arm of the Achilles tendon (*L*_Ach_) about the ankle joint, another is the ratio between the length of the forefoot and *L*_Ach_ (Scholz et al., 2008; Spurrs et al., 2003). The size of the *L*_Ach_ is highly determined by the size of the calcaneus bone and is regarded as a highly specialized feature of the human species for the evolution of *Endurance Running* and *Persistence Hunting* (PH) in the genus *Homo* (Raichlen et al., 2011). It is speculated that hominids during PH ran at speeds that forced animals to enter hyperthermia (Pontzer et al., 2009).

Despite the remarkable differences in RE between runners, it is largely unknown, which factors are decisive for a high RE (low VO_2_ kg^−1^ at a specific velocity). Since 1968, African and especially Kenyan runners have dominated the international scene in middle‐ and long‐distance races to a degree that has been termed the greatest geographical concentration of sports excellence in the annals of sports (Larsen & Sheel, 2015). Accordingly, the Kenyan runners have been subjected to research projects regarding their anatomy, physiological capabilities, and biomechanical characteristics. Saltin et al. (Saltin, 2003) concluded that no differences between Kenyan and European runners could be observed regarding VO_2max_, muscle fiber type distribution, number of capillaries or metabolic enzymes (Saltin, Kim et al., 1995; Saltin, Larsen et al., 1995). Biomechanically, only contact time has been reported shorter in Kenyan runners (Santos‐Concejero et al., 2017). Regarding anatomical differences, it has been reported that elite Kenyans had longer shanks and longer Achilles tendons than Japanese elite runners (Kunimasa et al., 2014; Sano et al., 2015). However, the Achilles tendon moment arm (*L*_Ach_) was found to be longer in Kenyan than Japanese elite runners (Kunimasa et al., 2014), which is contradictory to studies reporting significant correlations between *L*_Ach_ and RE (Barnes et al., 2014; Scholz et al., 2008) showing a positive effect of a short *L*_Ach_. Due to these discrepancies, it was decided to measure the *L*_Ach_ of the athletes in the present study and reinvestigate any possible correlation with RE.

It seems obvious that RE somehow should relate to “running technique,” but no studies have been able to show a relation between the movement pattern of middle‐ and long‐distance running and RE. Within *Track and Field Athletics* it is well known that changing the movement pattern of a distance runner is “dangerous” and will often result in impaired performance. Most often runners successfully choose their step frequency and stride length from subjective criteria, which was shown already by Högberg (1952). Accordingly, one purpose of the present study was to relate biomechanical calculations of mechanical energy during running to RE in elite middle‐ and long‐distance runners.

Lower leg thickness has been found to correlate significantly to RE and especially for Kenyan runners, who were claimed to have more slender legs than European runners (Saltin, 2003). Based on this finding it was suggested that it would be less energy demanding to move a lower leg mass back and forward during the swing phase of running (Larsen et al., 2004; Saltin, 2003). It was therefore decided to measure foot and lower leg volume of the athletes in the present study to see if this anatomical parameter would be significantly correlated with RE.

Muscular fascicle length has been shown to correlate significantly with maximal sprint running speed and it was suggested that longer muscle fibers would infer a more beneficial force–velocity relationship of the leg muscles (Abe et al., 2001). As this mechanism also could cause the muscles to produce the same muscle force at a lower contraction velocity and thereby the use of fewer muscle fibers at a given running velocity, it was decided to measure muscular fascicle length by use of ultrasonography and relate this parameter to RE.

## 1 METHODS

### 1.1 Subjects

Twelve elite, male, middle‐ and long‐distance runners (Table 1) gave their voluntary consent to participate in the study. The athletes competed at national or international level in events ranging from 800 m to 10 km. Characteristics of the subjects are presented in Tables 1 and 2. The protocol was approved by the Research Ethics Committee for Science and Health, University of Copenhagen, Denmark.

**TABLE 1:** Subject data

	Height (m)	Weight (kg)	BMI	Age (y)	VO_2max_
Mean	1.82	68.5	20.54	22.4	67.0
SD	0.06	7.66	1.21	3.1	4.2

**TABLE 2:** Personal best results of the athletes

Athlete	800 m	1500 m	5000 m	10.000 m
1	1.53.99 min	3.41.17 min		
2	1.51.11 min	3.53.35 min		
3				31.03.00 min
4				30.45.00 min
5	1.58.17 min	3.57.92 min		
6	1.57.73 min	3.57.69 min		
7	1.54.00 min	3.53.37 min		
8	1.49.44 min	3.49.59 min		
9		4.06.26 min	14.51.25 min	
10		4.04.13 min		
11	1.55.55 min	3.49.44 min		
12	1.49.50 min			

### 1.2 Experimental protocol

The subjects visited the laboratory on 3 consecutive days. On day 1, a treadmill test was completed to determine running economy (RE) and VO_2max_. On day 2, anthropometric and muscular variables were determined. On day 3, biomechanical variables related to running were determined.

### 1.3 Running economy and VO_2max_

Running Economy was determined as the rate of oxygen consumption (VO_2_) per kg body mass while running at two different submaximal velocities on a motorized treadmill (Woodway Desmo Pro Treadmill, Woodway Inc). The speed of 14 km h^−1^ was chosen as a “safe” velocity with regard to the expected aerobic capacity of the athletes. The speed of 18 km h^−1^ was chosen to represent a velocity close to the conditions during competition. After a standardized warm up on the treadmill, the subjects ran at two submaximal running speeds 14 and 18 km h^−1^, 0% grade, for 4 min separated by 1–2 min rest. During the 4‐min stages, in and expired gases were measured continuously by a gas analyzer (MasterScreen CPX, CareFusion). Breath‐by‐breath data were processed by the software system JLab (CareFusion). Running economy (RE) was determined as the mean VO_2_ (ml kg^−1^ min^−1^) during the last minute of each 4‐min bout.

A few minutes after the last submaximal run, an incremental test to exhaustion was completed to determine VO_2max_. The test started at 16 km h^−1^ and the speed was increased by 1 km h^−1^ each minute until 20 km h^−1^. After a minute at this velocity, the treadmill gradient was increased by 1% each minute until exhaustion. VO_2max_ was determined as the highest mean VO_2_ over a 30 s period. Values of VO_2_expressing resting values were obtained from the difference between VO_2_at 14 and 18 km h^−1^ divided by 4 km h^−1^. These values were subtracted from all the measured values of VO_2_.

### 1.4 Anthropometric measurements

**FIGURE 1:**
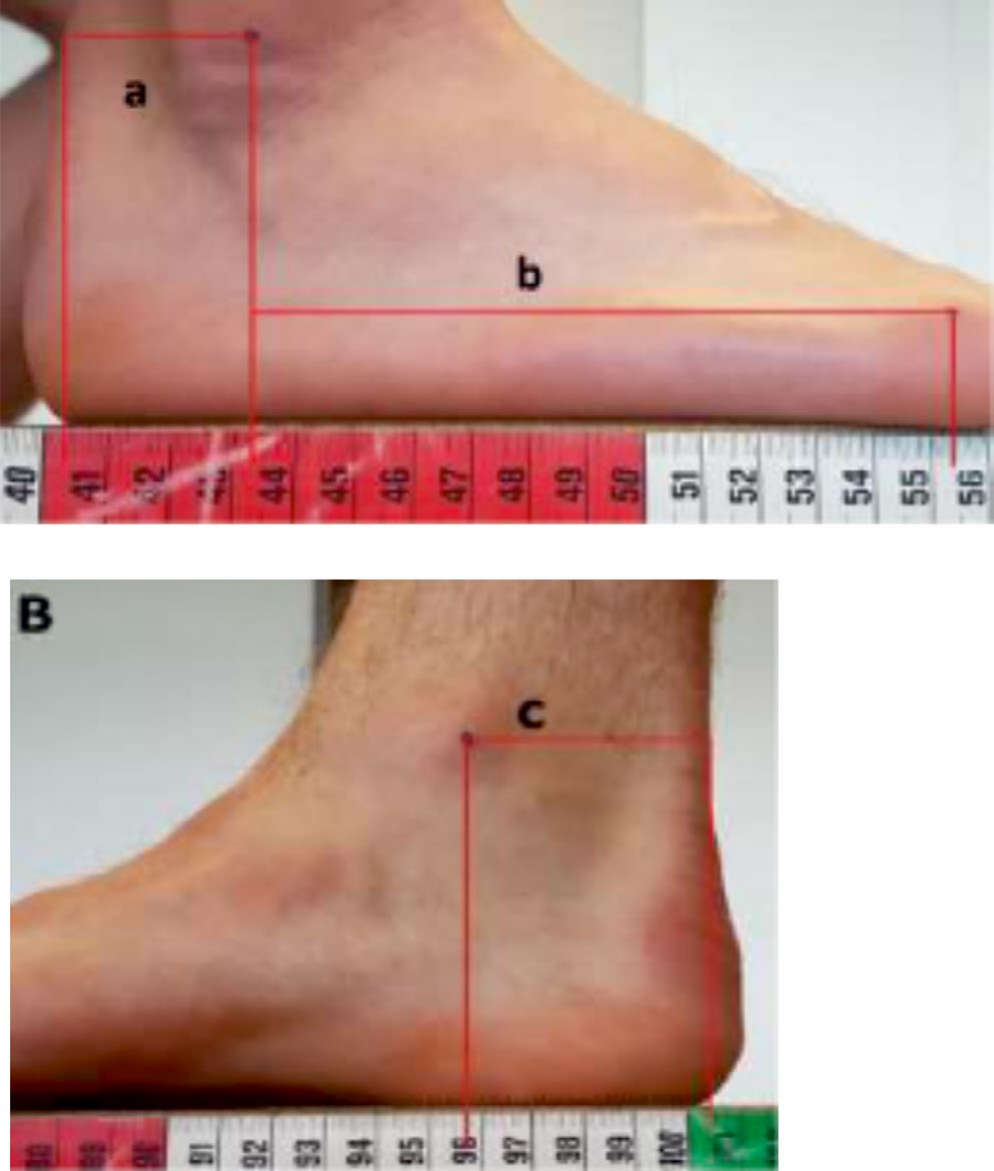
The lateral Achilles tendon moment arm (a) (top) and (bottom) the medial Achilles tendon moment arm (c). The resulting Achilles tendon moment arm (*L*_Ach_) was calculated as the mean of a and c. The length of the forefoot is shown as distance b

The subjects’ Achilles tendon moment arm (*L*_Ach_) was measured by the method presented by Scholz et al. (2008). Briefly, the most prominent part of the lateral and medial malleolus of the subjects’ right foot was marked. The subjects were seated in a chair with their foot on a reference block. First, foot and leg were positioned so that the lateral edge of the foot was aligned with the reference block and the anterior border of the tibia was vertical. From this position the lateral side of the foot and leg was photographed (Figure 1). The same procedure was used for the medial side. The medial edge of the foot was aligned with the reference block, the anterior border of the tibia was vertical, and the medial side was photographed (Figure 1). The horizontal distance from the marked spot on the malleolus to the posterior aspect of the Achilles tendon was measured on the pictures. This was performed for both the lateral and medial sides, and the *L*_Ach_ was determined as the mean of two values.

From the picture of the lateral side, the length of the forefoot was also determined by measuring the horizontal distance from the marked spot on the lateral malleolus to the head of the fifth metatarsal (marked by a spot). The length of the forefoot was determined as the mean of two consecutive measurements.

Lower leg and foot volume were determined by scanning the lower leg and foot with a hand‐held 3‐D surface scanner (Artec Eva, Artec 3D, Luxembourg) (Tierney et al., 1996). Proximally, the lower leg was marked by two markers, one on caput fibula and one on tibia at about equal heights. Distally, the lower leg was marked by two additional markers, one on the lateral malleolus (of tibia) and one on the medial side at about equal heights (approximately 1 cm below the medial malleolus). This procedure was used on both the right leg and the left leg. The distal markers on the lower legs were used to mark the feet as well.

During the scanning of the lower legs, the subjects were instructed to stand in a relaxed upright position with enough space between the feet for the scanner to be able to scan the medial side of the lower legs. Tape was used to mark the subject's foot position to guarantee accuracy during and between the measurements. A minimum of three scans were applied to the lower legs.

During the scanning of the feet, the subjects sat in a chair with their right lower leg resting on another chair, so that the foot was free from the chair. The subjects were instructed to relax their foot during all scans and the lower leg was fastened with sports tape, so that movement of the lower leg and foot was minimized. This was repeated for the left leg, and a minimum of three scans were performed on each foot.

A 3‐D model was constructed and further processed using Artec Studio (Artec 3D, Luxembourg). The lower legs were isolated from the 3‐D model by cutting off everything proximally and distally to the two marker pairs, respectively. The feet were isolated by cuts proximal to the distal markers. The volumes of the 3‐D models of the isolated lower legs and feet were calculated using the Artec Studio software. The volumes of two successful scans of each lower leg were calculated, and the volume of the lower leg was determined as the mean of these. The same procedure was applied to calculate the volume of the foot.

Body mass and height were measured using standard procedures and, in addition, the following anthropometric variables on the subjects’ right side were determined: total leg length (from the ground to spina iliaca anterior superior), thigh length (from trochanter major to the lateral condyle of the femur), shank length (from caput fibulae to the lateral malleolus of the tibia), foot length (from the back of the heel to the tip of the longest toe), forefoot length from the lateral malleolus to the fifth metatarsal joint (Figure 1), and toe length (from the head of the first metatarsal to the tip of the first phalanx distalis).

### 1.5 Fascicle length

Fascicle length (*L*_f_) was estimated using a B‐mode ultrasound scanner (LS128, CEXT‐1Z, Telemed Ltd.) and transducer (LV8‐5L60N‐2 veterinary, Telemed Ltd.). The vastus lateralis (VL), gastrocnemius medialis (GM), and soleus (SOL) muscle of the subject's right leg were scanned. For VL, the transducer was placed at a point midway between trochanter major and the lateral condyle of the femur. For GM and SOL, the transducer was placed at a point approximately 30% proximally between the medial condyle and the medial malleolus of the tibia and midway between the medial and lateral borders of the GM (Abe, 2002). During the scans, the subjects stood in an upright relaxed position and the transducer was placed parallel to the muscle fibers and adjusted if necessary to get the optimal picture. The ultrasound images of the muscles were recorded by Echo Wave II software (3.4.0, Telemed Ltd.). The fascicle pennation angle (*α*) was determined as the angle between the deep aponeurosis and the fascicles of the specific muscle (Abe et al., 2000; Cronin & Lichtwark, 2013; Kawakami et al., 2002). The isolated muscle thickness (*T*_m_) was determined by measuring the distance between the deep and superficial aponeurosis of the specific muscle (Aggeloussis et al., 2010). This was performed for both the proximal and distal ends of the muscle visualized in the ultrasound image, and a mean of these two distances was used as *T*_m_. The *L*_f_ was estimated using the following equation:

$$L_{f}=\frac{T_{m}}{\sin(\alpha )}$$

The *L*_f_ of each muscle was determined as a mean of three estimated *L*_f_ of the specific muscle and expressed both in absolute values (cm) and relative to the related segment length (cm cm^−1^).

### 1.6 3‐D biomechanical movement analysis

**FIGURE 2:**
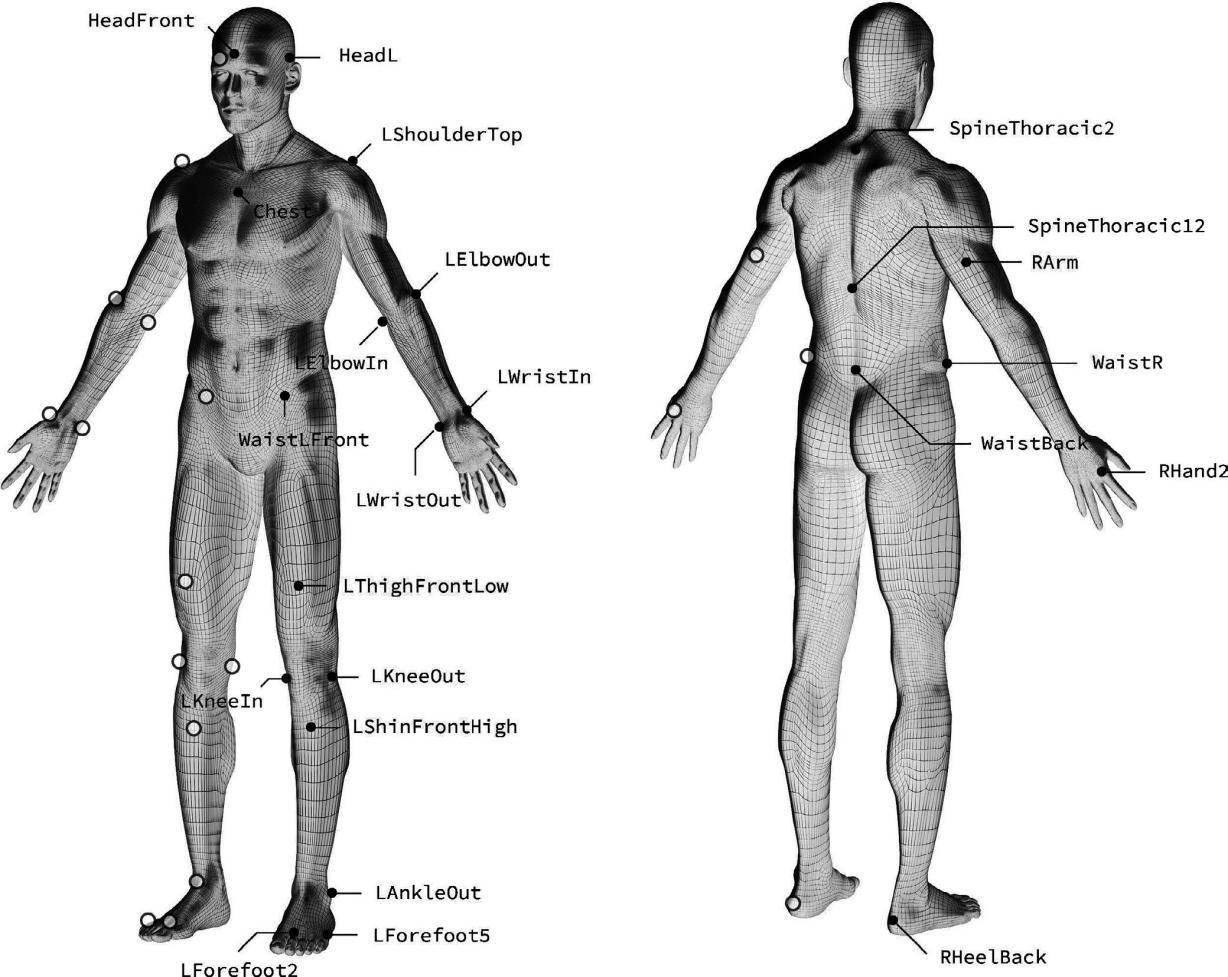
Reflective spherical markers were placed at anatomical landmarks. Reproduced with permission of Qualisys AB

Due to injuries (not related to this study), only 10 of the 12 subjects managed to complete a 3‐D biomechanical analysis of treadmill running to determine stride frequency (*f*_s_), stride length (*L*_s_), contact time (*t*_c_), swing time (*t*_s_), vertical oscillations, and mechanical work. Thirty‐five spherical reflective markers were placed on selected anatomical landmarks (Figure 2). After a standardized warm up, the subject ran at the two submaximal velocities from the RE protocol (14 and 18 km h^−1^, 0% grade) while recorded by a Qualisys system for movement analysis (Qualisys AB). Eleven high‐speed infrared cameras (300 Hz) recorded a minimum of 15 steps at each velocity. Three‐dimensional coordinates of the markers were exported to the software system AnyBody (AnyBody version 7.1, AnyBody Technology A/S), which was used to analyze the recordings. Simultaneously, the athletes were recorded on video (120 frames s^−1^) and these recordings were later used to obtain contact time, stride frequency, swing time, and stride length. Stride length was calculated as velocity divided by stride frequency.

AnyBody is a multibody dynamics system, which discretizes the body into links representing the bones as rigid segments articulating at the anatomical joints. To each bone was assigned the mass of the other tissues surrounding the bone, such that the sum of segment masses equaled the total body mass and the distribution of masses followed Dempster (Dempster, 1955).

The potential energy of the system was computed as the sum of potential energies of the segments. Similarly, the kinetic energy was computed as the sum of segment kinetic energies, where each segment's kinetic energy contained translational and rotational contributions (Winter, 1979). The mechanical energy of each segment and of the entire system, *E*_mech_, was calculated as the sum of potential and kinetic energy (Winter, 1979).

During motion, energy is converted between kinetic and potential contributions, existing energy is exchanged between segments via joint reaction forces and muscle connections, and energy is produced or dissipated by positive and negative muscle work in a complex interplay. The internal exchange of energy between segments is complicated, but disregarding friction, air resistance, and other dissipative effects, the net change in mechanical energy of the entire system is attributed to muscle work (Winter, 1979). We therefore defined the mechanical muscle power of the whole system as follows:

$$P_{\text{mech}}=\frac{d\;E_{\text{mech}}}{\mathrm{dt}}$$

*P*_pos_ and *P*_neg_ were defined as the sum of the positive and negative increments in *P*_mech_, respectively.

Subsequently, we computed the metabolic power as

$$\begin{array}{c} P_{\text{metab}}=\left\{\begin{array}{c} P_{\text{mech}}/0.25\;\text{if}\;P_{\text{mech}}\geq 0\\ P_{\text{mech}}/‐1.20\;\text{if}\;P_{\text{mech}}<0 \end{array}\right. \end{array}$$

that is, different metabolic efficiencies for concentric and eccentric muscle work (Aura & Komi, 1986; Laursen et al., 2000). When expressing the mechanical work intensity as liter O_2_ min^−1^, an energetic value of 20 kJ per liter oxygen was used. A measure of gross efficiency was obtained by dividing *P*_mech_ by *P*_metab_.

### 1.7 Stiffness

Stiffness of the whole body was measured during running as previously described (Cavagna et al., 1977; Ferris et al., 1998; McMahon & Cheng, 1990; Morin et al., 2005). The vertical ground reaction force was calculated in the AnyBody system by the methods described by Fluit et al. (2014) and by Skals et al. (2017). The vertical trajectory of the body center of mass (BCM) was also computed by the AnyBody system using anthropometrics from Dempster (1955). Thus, the vertical stiffness *k*_vert_ in kN m^−1^ was calculated by the formula:

$$k_{\text{vert}}=\frac{F_{\max}}{\Delta y}$$

where Δ*y* is the vertical displacement of BCM from touch down (heel strike) till *F*_max_, which is the peak value of the vertical ground reaction force.

Leg stiffness of the support leg during running was calculated by the formula:

$$k_{\text{leg}}=\frac{F_{\max}}{\Delta L}$$

where:

$$\Delta L=L‐\sqrt{L^{2}‐{\left(\frac{v\cdot tc}{2}\right)}^{2}}+\Delta y$$

where *L* is leg length and Δ*y* is the vertical displacement of the body center of mass at its lowest point during the contact phase. It has been shown that BCM is at its lowest point at the time of *F*_max_ (Morin et al., 2005). At each running velocity, stiffness was measured in three consecutive steps and averaged.

### 1.8 Statistics

Spearman's rank correlation analysis was used to determine the relationship between RE and the anthropometric, biomechanical, and muscular variables of the subjects (Matlab R2018a, The MathWorks Inc). The level of significance was set to *p* < 0.05.

## 2 RESULTS

Personal data and VO_2max_ of the athletes are listed in Tables 1 and 2. The group mean value of VO_2max_ was 67.0 ml O_2_ kg^−1^ (range: 61.7–78.2), which confirmed that the athletes were all well‐trained elite runners (Table 3).

**TABLE 3:** Running economy at 14 and 18 km h^−1^, respectively

Athlete	VO_2_ ml kg^−1^ min^−1^ 14 km h^−1^	VO_2_ ml kg^−1^ km^−1^ 14 km h^−1^	VO_2_ ml kg^−1^ min^−1^ 18 km h^−1^	VO_2_ ml kg^−1^ km^−1^ 18 km h^−1^	VO_2max_ ml kg^−1^ min^−1^	% VO_2max_ 14 km h^−1^	% VO_2max_ 18 km h^−1^
1	45.3	194	58.2	194	65.4	69.3	89.1
2	47.9	205	62.2	207	68.6	69.7	90.6
3	39.8	170	54.5	182	68.0	58.5	80.2
4	44.2	189	57.5	192	66.3	66.7	86.7
5	45.7	196	65.7	219	78.2	58.5	84.1
6	47.3	202	61.1	204	67.4	70.1	90.6
7	42.9	184	59.8	199	66.0	65.0	90.7
8	40.9	175	57.0	190	61.7	66.2	92.3
9	41.4	177	54.4	181	69.1	59.9	78.7
10	48.6	208	60.2	201	64.7	75.1	93.1
11	43.1	185	56.3	188	65.7	65.6	85.8
12	42.4	182	57.7	192	62.4	67.9	92.4
Mean	44.1	189	58.7	196	67.0	66.1	87.9
SD	2.9	12.3	3.3	10.9	4.2	5.0	4.8

Resting values calculated from the difference between VO_2_ at the two running velocities were on average 3.66 ml O_2_ kg^−1^ min^−1^ (±0.60). Running economy (RE) corrected for resting values was (averaged across subjects) 44.1 and 58.7 ml O_2_ kg^−1^ min^−1^ at 14 and 18 km h^−1^, respectively (Table 3). This implied at 14 km h^−1^ a difference of 22% and at 18 km h^−1^ a difference of 21% between the best athlete and the poorest athlete. RE at 14 and 18 km h^−1^ was significantly correlated (Rho = 0.79, *p* = 0.0021) indicating a linear relationship between RE and running velocity as shown before (Saltin, Kim, et al., 1995; Saltin, Larsen, et al., 1995; Saunders et al., 2004a, 2004b).

Without correction for resting metabolism, RE was on average 47.8 (±2.8) ml O_2_ kg^−1^ min^−1^ and 62.4 (±3.6) ml O_2_ kg^−1^ min^−1^ at 14 and 18 km h^−1^, respectively. Uncorrected VO_2max_ was 70.8 (±4.7) O_2_ kg^−1^ min^−1^.

The relative load of the athletes at 14 and 18 km h^−1^ was on average 66.1% (±5.0) and 87.9% (±4.8) with respect to VO_2max_ (Table 3).

Biomechanical and temporal parameters related to the step cycle (step rate, step length, contact time, swing phase, and BCM oscillations) were not correlated with RE (Table 4).

**TABLE 4:** Running step parameters. “BCM oscillations” are body center of mass vertical oscillations. No significant correlations between these parameters and running economy were observed

	Step rate	Step length	Contact time	Swing phase	BCM oscillation
14 km h^−1^	2.82 (Hz) (0.12)	1.38 (m) (0.06)	171 (ms) (9.22)	541 (ms) (31.7)	8.8 (cm) (1.2)
18 km h^−1^	2.96 (Hz) (0.09)	1.70 (m) (0.05)	138 (ms) (10.4)	542 (ms) (31.7)	8.3 (cm) (1.0)

**FIGURE 3:**
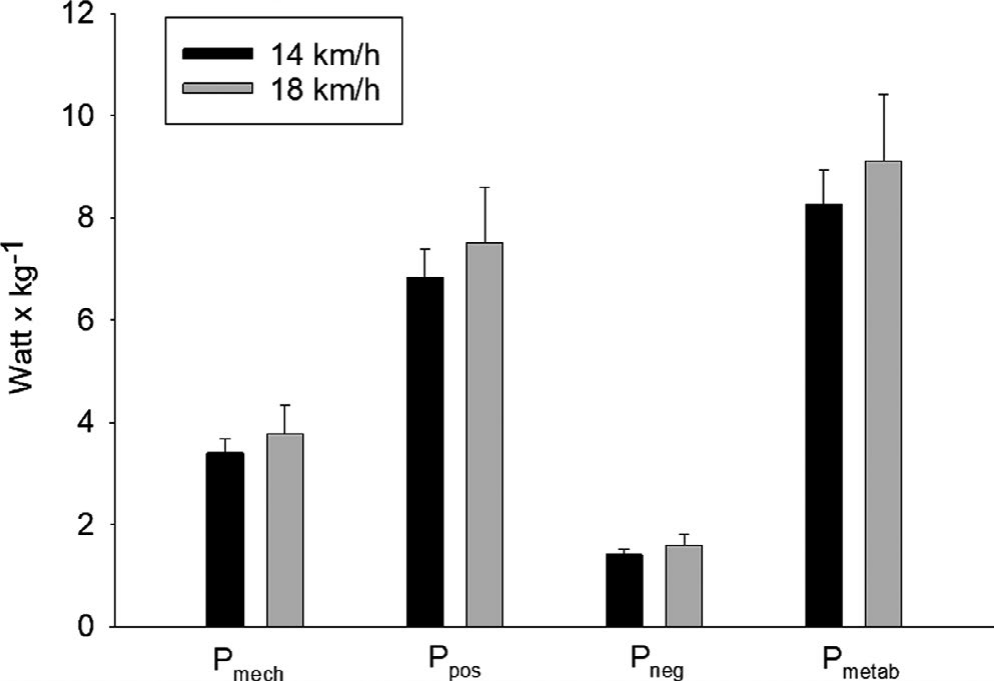
Mechanical work intensity *P*_mech_. The positive (*P*_pos_), negative (*P*_neg_), and the metabolic calculated work (*P*_metab_) are corrected by 25% efficiency for positive work and −120% for negative work. Error bars are one standard deviation

The mechanical work intensity (*P*_mech_) was 3.41 (0.28) and 3.79 (0.54) W kg^−1^ for 14 and 18 km h^−1^, respectively (Figure 3). None of the parameters expressing mechanical work intensity were significantly correlated with RE. However, body mass was significantly correlated with VO_2_ (L min^−1^) at 14 km h^−1^ (Rho = 0.89, *p* = 0.0014) and at 18 km h^−1^ (Rho = 0.93, *p* = 0.0001). Body mass was also significantly correlated with *P*_mech_ at 18 km h^−1^ (Rho = 0.71, *p* = 0.0275).

**FIGURE 4:**
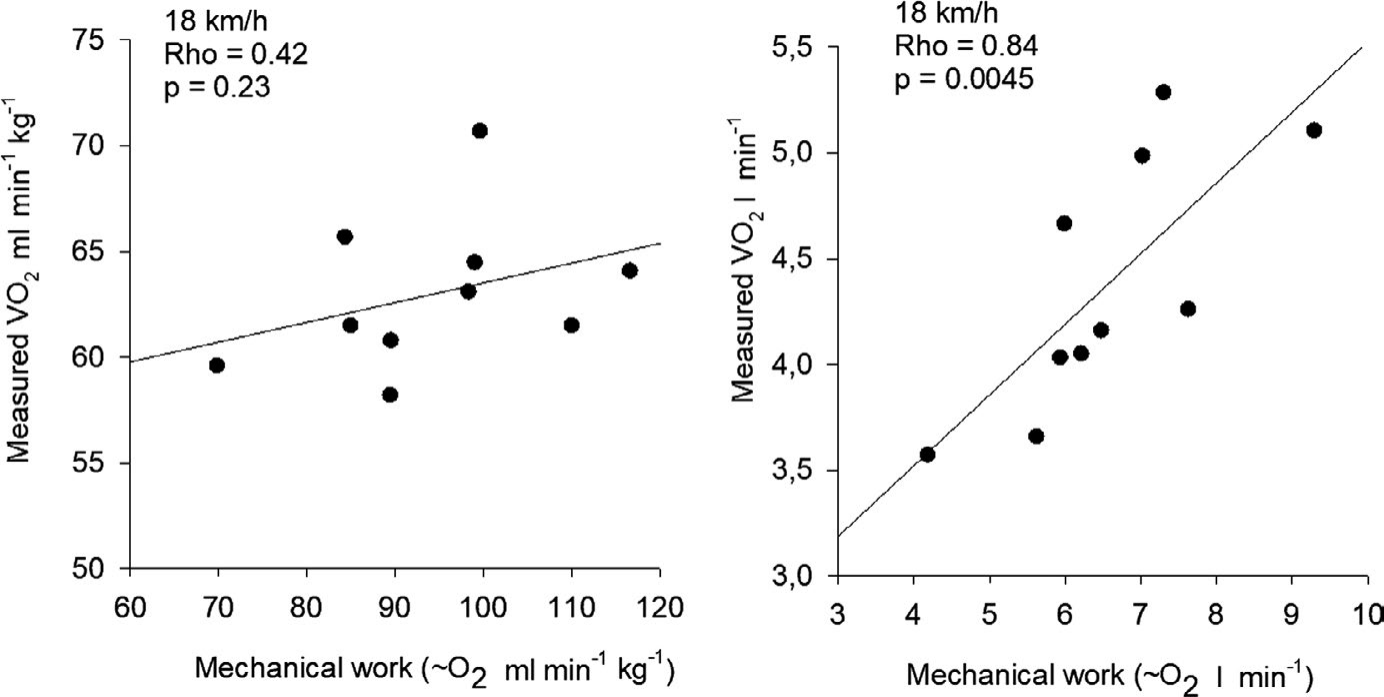
Left: Relation between mechanical work expressed as ml O_2_ min^−1^ kg^−1^ and measured VO_2_ min^−1^ kg^−1^ at 18 km h^−1^. Right: Relation between mechanical work expressed as liter O_2_ min^−1^ and actually measured VO_2_ at 18 km h^−1^

When the mechanical work intensity was expressed as liter O_2_ min^−1^, significant correlations were found between mechanical work intensity and the measured VO_2_ in L min^−1^ (Figure 4). At 14 km h^−1^, Rho was 0.66 (*p* = 0.044) and at 18 km h^−1^ Rho was 0.84 (*p* = 0.0045) (Figure 4). Respiratory quotient ratio (RER) values were 0.85 (range: 0.68–0.93) and 0.935 (range: 0.76–1.04) for 14 and 18 km h^−1^, respectively.

Gross efficiency calculated on mechanical data only was 41.4% and 41.9% at 14 and 18 km h^−1^, respectively.

*P*_mech_ at 14 and 18 km h^−1^ was significantly correlated (Rho = 0.68, *p* = 0.055) as was *P*_neg_ (Rho = 0.66, *p* = 0.044).

**FIGURE 5:**
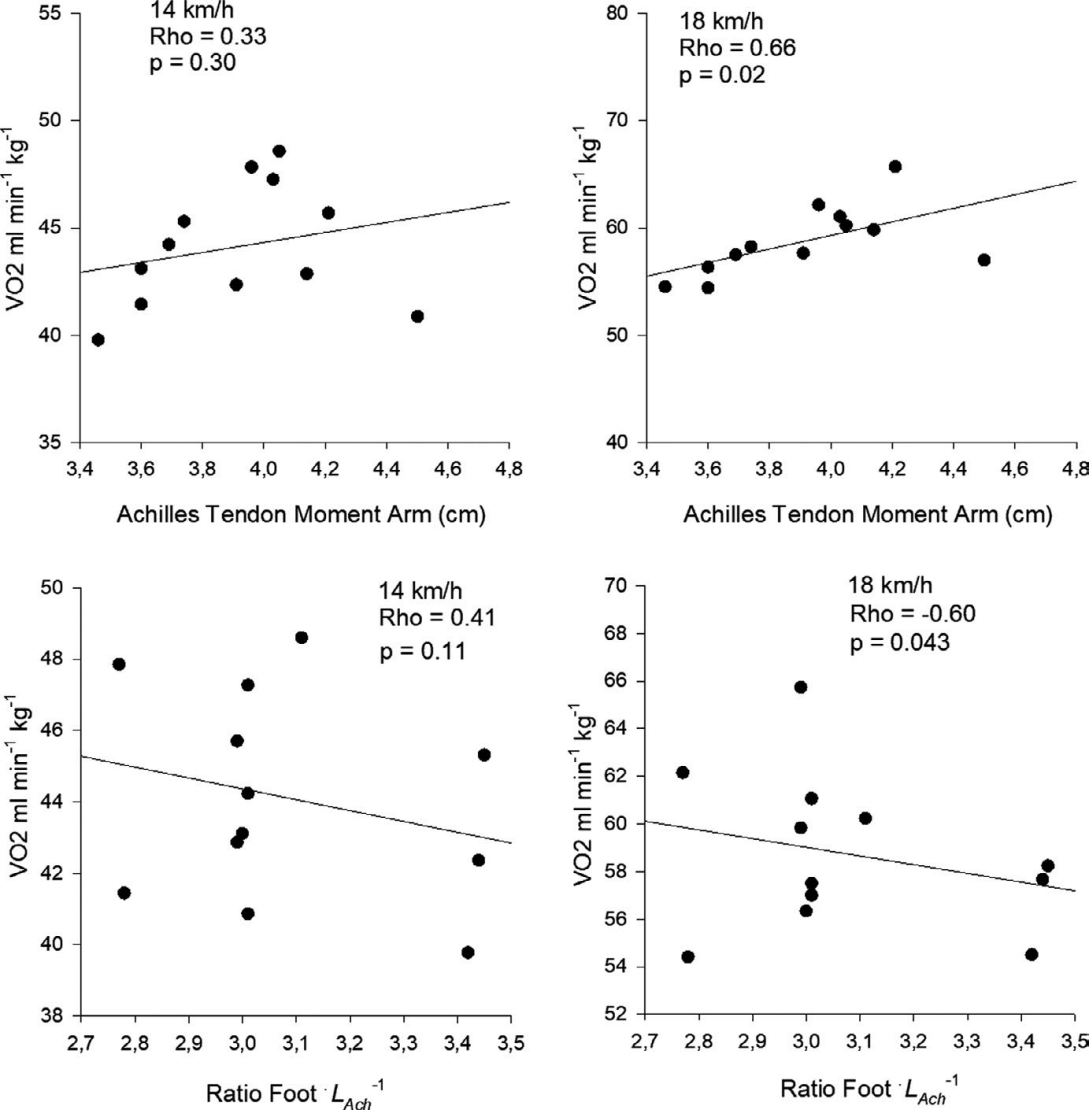
Relation (top) between Achilles tendon moment arm (*L*_Ach_) and RE and (bottom) relation between foot ratio and RE. Foot ratio is forefoot *L*_Ach_^−1^

The Achilles tendon moment arm (*L*_Ach_) was on average 3.91 cm and was significantly correlated with RE at 18 km h^−1^ (Rho = 0.73; *p* = 0.007) (Figure 5) (Table 5). This implied that a short moment arm is an advantage regarding RE at 18 km h^−1^ while not at 14 km h^−1^. The *L*_Ach_ varied from 3.46 to 4.21 cm corresponding to a difference of 17.8% between the extremes of the group (Table 5; Figure 5).

**TABLE 5:** Soleus moment arm, leg (shank) and foot volumes. * denotes a significant correlation to running velocity at 18 km h^−1^ (Rho = −0.66; *p* = 0.02)

Athlete	Achilles tendon moment arm (cm)	Shank volume (liters)	Foot volume (liters)
1	3.74	3.59	1.24
2	3.96	3.01	1.00
3	3.46	2.78	0.81
4	3.69	2.93	0.89
5	4.21	2.78	0.92
6	4.03	2.28	0.77
7	4.14	2.71	0.97
8	4.50	2.65	0.90
9	3.60	2.39	0.83
10	4.05	2.64	0.88
11	3.60	2.01	0.76
12	3.91	2.49	0.85
Mean	3.91*	2.69	0.90
SD	0.30	0.40	0.13

**FIGURE 6:**
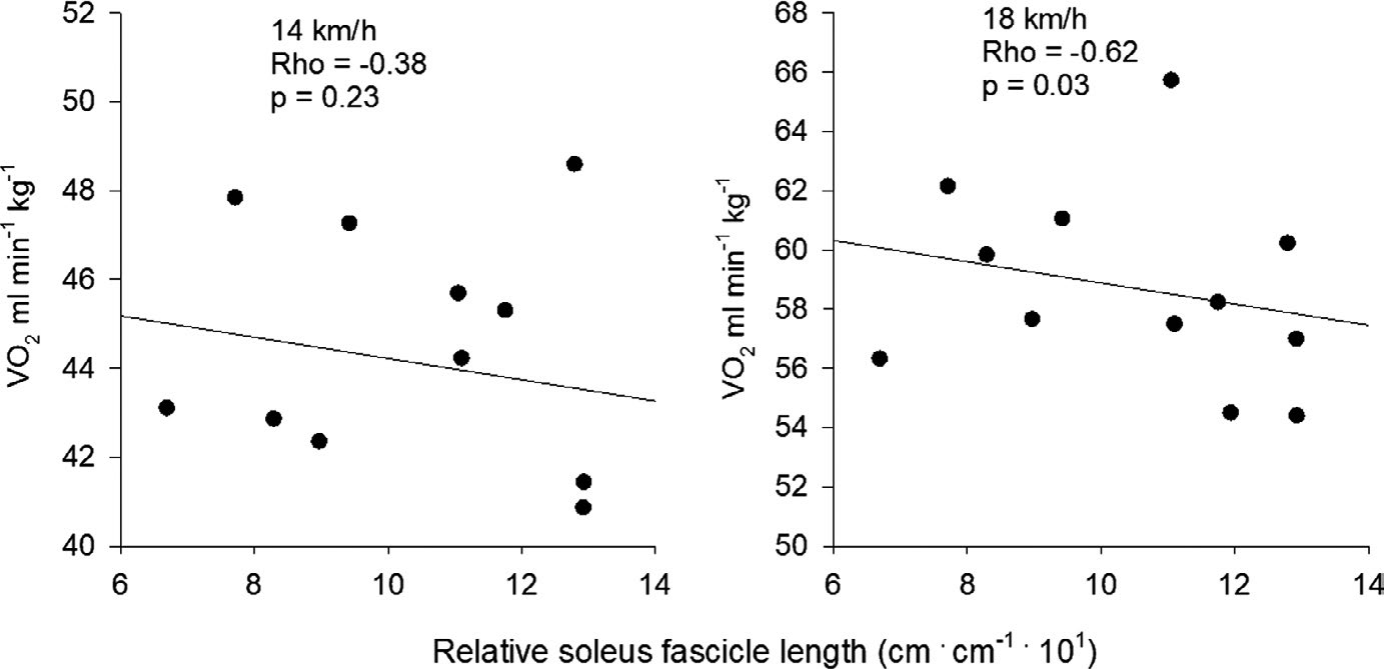
Relation between RE and relative soleus fascicle length at 14 and 18 km h^−1^. The correlation at 18 km h^−1^ was statistically significant (Rho = −0.62; *p* = 0.03)

Fascicle length of m. soleus (SO) was 4.1 cm on average and varied from 3.2 to 4.9 cm corresponding to a 36% difference between the subject with the shortest and the subject with the longest fascicles. Similar differences were observed for the gastrocnemius (GM) (mean 5.6 cm; range: 4.5–6.8) and the vastus lateralis (VL) (mean 6.6 cm; range: 5.6–7.9). Individual range for the GM corresponded to 34% and for the VL 29%. No significant correlations were found between absolute fascicle length and RE, neither at 14 nor at 18 km h^−1^. However, when normalized to leg (shank) length the soleus fascicles showed a significant correlation with RE at 18 km h^−1^ (Rho = −0.62; *p* = 0.03) (Figure 6).

Total leg length, shank length, foot length, and toe length were not significantly correlated with RE (Table 5). The same was the case for shank and foot volumes (Table 5). However, the foot ratio between the forefoot and the Achilles tendon moment arm was significantly correlated with RE at 18 km h^−1^ (Rho = −0.64; *p* = 0.030) (Table 5) (Figure 5), that is, a greater ratio seems an advantage regarding RE.

**FIGURE 7:**
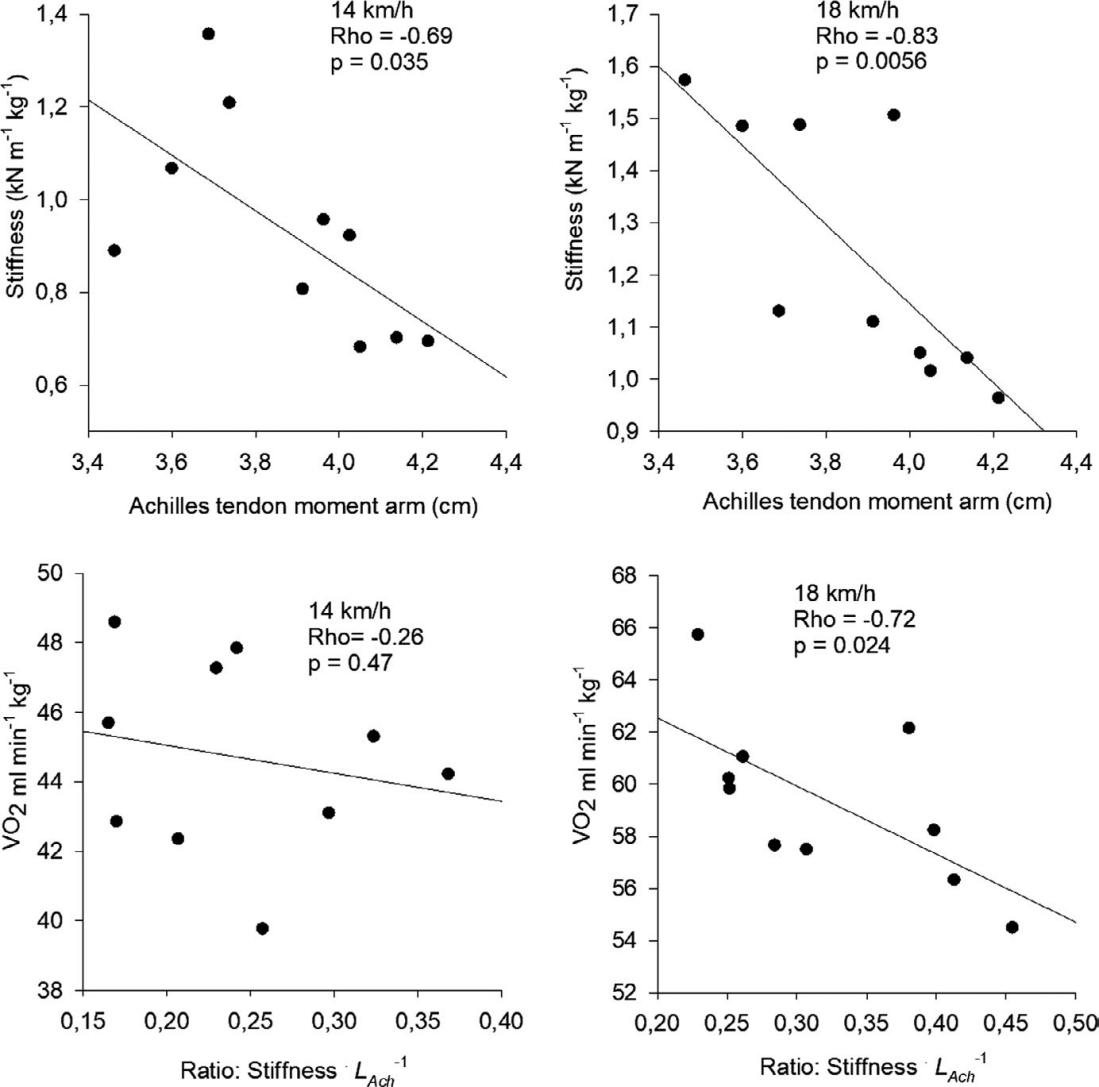
On top: relation between whole body stiffness and Achilles tendon moment arm. Bottom: relation between RE and the ratio between stiffness and Achilles tendon moment arm

Whole body stiffness normalized to body mass was 930 (±227) N m^−1^ kg^−1^ at 14 km h^−1^ and 1240 (±240) N m^−1^ kg^−1^ at 18 km h^−1^. Leg stiffness (*k*_leg_) was 900 (±220) and 1200 (±230) N m^−1^ kg^−1^. None of these stiffnesses were significantly correlated with RE (Rho = −0.18, *p* = 0.63 and Rho = −0.58, *p* = 0.088, respectively). Whole body stiffness at 14 km h^−1^ (Rho = −0.69; *p* = 0.035) and at 18 km h^−1^ (Rho = −0.75; *p* = 0.018) was significantly correlated with the Achilles tendon moment arm (*L*_Ach_) (Figure 7) indicating that a short moment arm coincided with high stiffness. Also leg stiffness was significantly correlated with the Achilles tendon moment arm at 14 km h^−1^ (Rho = −0.7; *p* = 0.025) and at 18 km h^−1^ (Rho = −0.83; *p* = 0.006). The ratio between whole body stiffness and *L*_Ach_ was significantly correlated with RE at 18 km h^−1^ (Rho = −0.72, *p* = 0.024) (Figure 7). Absolute whole body stiffness (N m^−1^) was significantly correlated with body mass (Rho = 0.68, *p* = 0.035) and to absolute VO_2_ (L min^−1^) (Rho = 0.71, *p* = 0.028).

## 3 DISCUSSION

### 3.1 Mechanical power

By use of 2‐D biomechanical movement analysis, it has earlier been attempted to quantify mechanical power exerted by the muscles during human running. However, different approaches have been used as the mechanical work may be defined and/or divided into external work on the surroundings and internal work due to the movements of segments like arms, legs, and trunk. The external work has been measured by force platforms (Cavagna et al., 1976), accelerometers (Cavagna et al., 1964), or by movements of the center of mass of the whole body (Luhtanen & Komi, 1978). The internal work is calculated by summation of potential and kinetic energy of all body segments (Laursen et al., 2000; Winter, 1979). By use of these different approaches power values of 556 W (Cavagna & Kaneko, 1977), 172 W (Norman et al., 1976), 931 W (Luhtanen & Komi, 1978), and 396 W (Williams & Cavanagh, 1983) have been reported. These studies were based on 2‐D cinematography except the study of Williams and Cavanagh (Williams & Cavanagh, 1983), which was three dimensional with running velocities varying between 3.6 and 3.9 m s^−1^ (13–14 km h^−1^). Williams and Cavanagh subdivided their subjects into three groups based on RE at 3.57 m s^−1^ (approximately 13 km h^−1^) and observed a trend between relative positive power and three “Physiological Efficiency Groups” (Williams & Cavanagh, 1983) but, to the best of our knowledge, nobody has found a significant correlation between biomechanical calculations of power and measured VO_2_ during running.

In the present study, a 3‐D modeling approach was applied to velocities of 14 and 18 km h^−1^ and the mechanical power was found to be 237 (30.3) and 264 (53.9) Watt corresponding to 3.41 (0.28) and 3.79 (0.54) W kg^−1^, respectively (Figure 3). When mechanical power was expressed as metabolic cost corresponding to liter O_2_ min^−1^ a significant correlation was found between the mechanical calculations and the measured VO_2_ (Figure 4), indicating that there is a mechanical explanation behind RE. However, since body mass was also highly correlated with VO_2_ measured in absolute values, it is possible that the correlation only reflects the fact that heavy subjects consume more oxygen and produce more mechanical energy.

It was remarkable that VO_2_ calculated from mechanical power was almost twice as high as the actually measured VO_2_. A fixed value of 20 kJ per liter O_2_ was used to “convert” power to VO_2_ but using the actually measured respiratory quotient ratios would only have changed the calculated VO_2_ a negligible degree. The most likely explanation for the high calculated values is that summation of segment energies cannot account for storage and reuse of elastic energy in the tendons. This energy should be subtracted, but there is no way we can calculate or estimate the size of it.

When mechanical power was normalized to body mass, no significant correlations were found regarding RE, which could be due to oxygen consumption not being linearly related to body mass in terms of physiology. This is a well‐known phenomenon and it has been suggested to use body mass^0.75^ (Bergh et al., 1991). However, even body mass^0.66^ did not improve the correlations of the present study. It is not straight forward to explain the missing correlation between RE and relative mechanical work rate, but it may be an inherent problem that most biomechanical methods use anthropometric tables, like Dempster (Dempster, 1955), to calculate segmental masses and moments of inertia. This is also the case for the method presented by Winter (1979), which was used in the present study. When these body parameters only vary with body mass and segment lengths, it is obvious that this causes individual subjects to become more identical and thereby more difficult to separate mechanically regarding RE. A future approach to relate biomechanical movement analysis to RE should deal with individual differences between the real body segments of the subjects as we found an extreme difference of 56% between the highest and the lowest shank volume in the present study (Table 5).

The method used by the present study and by Williams & Cavanagh (1983) was introduced by Winter (1979, 2009). It accounts for exchange of energy both between and within segments, but it cannot deal with storage and reuse of elastic energy in the muscle–tendon unit. The method allows for calculating the positive and the negative mechanical work separately and by assuming a mechanical efficiency for eccentric and concentric work it is possible to estimate a net efficiency for running only based on biomechanical movement analysis. In the present study, net efficiency was 41.6 (0.26) % and 41.9 (1.09) % for 14 and 18 km h^−1^, respectively. This corroborated the net efficiency of 44% reported by Williams & Cavanagh (1983) and it indicates that the mechanical efficiency of running is significantly higher as the approximately 25% efficiency of pure concentric muscle work (Asmussen, 1953; Asmussen & Bonde‐Petersen, 1974; Aura & Komi, 1986).

More simple biomechanical parameters like contact time, stride rate, and stride length have been investigated on numerous occasions and have rarely been found to have any influence on RE (Barnes et al., 2014). One study found a shorter contact time in Kenyan runners and argued that this would influence stiffness and the ability to store and reuse elastic energy (Santos‐Concejero et al., 2017). In the present study, no significant correlations between these parameters and RE were seen (Table 4).

### 3.2 Achilles tendon moment arm and RE

In the study of Scholz et al. (2008) a significant correlation (*r* = 0.75) was reported between running economy (RE) at 16 km h^−1^ and the Achilles tendon moment arm (*L*_Ach_). In the present study, a significant correlation (Rho = 0.66) was found between *L*_Ach_ and RE at 18 km h^−1^.

In an extensive study of RE, 63 runners (24 females, 39 males) of collegiate or national level were examined regarding RE and Achilles tendon moment arm (*L*_Ach_) (Barnes et al., 2014). For all subjects, *L*_Ach_ showed a very high and significant correlation (*r* = 0.90) with RE at 14 km h^−1^ implying that a short moment arm is advantageous regarding RE. The *L*_Ach_ was on average 4.4 cm for males and 3.5 cm for females with *r*‐values of 0.82 and 0.81 between RE and *L*_Ach_. Accordingly, males and females had the same RE despite differences in *L*_Ach_ (Barnes et al., 2014).

In a study of Kenyan and Japanese long‐distance runners by Kunimasa et al. (2014) it was found that the Kenyan runners had significantly longer *L*_Ach_ (4.46 cm) than the Japanese runners (4,07 cm). *L*_Ach_ of the Kenyans ranged from approximately 3.6–5.1 cm (Figure 3 in Kunimasa et al., (2014)) and, when both Kenyan and Japanese runners were pooled, a significant correlation (*r* = 0.55) was found between *L*_Ach_ and a performance index (International Athletics Amateur Federation). This indicated a long moment arm to be an advantage, but notably, RE was not measured directly (Kunimasa et al., 2014; Spiriev, 2011).

Considering the results of the present study with an *r*‐value of 0.66, and the previous results from the literature with even higher *r*‐values (Barnes et al., 2014; Scholz et al., 2008), it appears safe to conclude that *L*_Ach_ is highly correlated with RE despite the results of (Kunimasa et al. (2014).

### 3.3 Running economy

RE at 16 km h^−1^ was 48 ml O_2_ kg^−1^ min^−1^ in Scholz et al. (2008) corresponding to 182 ml O_2_ kg^−1^ km^−1^ while in the present study RE was 44 ml O_2_ kg^−1^ min^−1^ at 14 km h^−1^ corresponding to 189 ml O_2_ kg^−1^ km^−1^. This remarkable difference is difficult to explain. The maximal oxygen uptake was 67 ml O_2_ min^−1^ kg^−1^ in the present study but only 55 ml O_2_ min^−1^ kg^−1^ in Scholz et al. (2008), so it cannot be excluded that a systematic difference existed between the apparatus used for gas analysis during running, especially as the Dutch athletes were described as “highly trained” (Scholz et al., 2008). When RE is expressed as ml O_2_ kg^−1^ km^−1^, it is possible to compare RE at different running velocities. Accordingly, RE ranges from 170 to approximately 250 ml O_2_ kg^−1^ km^−1^ in the literature. The athletes of the present study ranged from 170 to 219 ml O_2_ kg^−1^ km^−1^ (Table 3) and the Olympic Champion Frank Shorter (USA, Olympic marathon winner, 1972) has been reported to have had a RE of 172 ml O_2_ kg^−1^ km^−1^ while Joseph Ngugi (Kenyan Olympic gold medalist on 5000 m, 1988) has been reported to have had a RE of 170 ml O_2_ kg^−1^ km^−1^ (Saltin, Larsen, et al., 1995). Besides the study of Scholz et al. (2008), one other study has reported very low values of VO_2_ during submaximal running (147–157 ml O_2_ kg^−1^ km^−1^) (Spurrs et al., 2003) and correspondingly low values of VO_2max_ (< 60 ml O_2_ min^−1^ kg^−1^). In fact, the runners in Spurrs et al. (2003) appeared to have a RE better than the best Kenyan and African runners ever measured (Larsen, 2003; Larsen & Sheel, 2015; Saltin, 2003; Saltin, Larsen, et al., 1995; Weston et al., 2000), which is highly unlikely.

### 3.4 Foot lever ratio

Kunimasa et al. found a significant correlation between IAAF score (Spiriev, 2011) and a ratio between the forefoot and the *L*_Ach_ (Kunimasa et al., 2014). It turned out that the Kenyans had a shorter forefoot and longer *L*_Ach_ than the Japanese runners. A contradictory and significant correlation was found between the same foot ratio and RE at 18 km h^−1^ in the present study (Figure 5). A possible explanation for this could be that RE was not measured in the study of Kunimasa et al. (2014) as an IAAF score was used instead. The foot lever ratio is an interesting property as it has been suggested that a certain gear ratio between the active muscles and the moment arm of the external ground reaction force may affect the energy cost of locomotion (Biewener et al., 2004; Carrier et al., 1994; Karamanidis & Arampatzis, 2007).

### 3.5 Leg volume

A strong relation has been reported between RE and lower leg circumference, which further indicated a trend toward Kenyan elite runners having a lower (smaller) leg thickness than European runners (Saltin, 2003), and it has been suggested that lighter shanks could partly explain the superior RE observed in Kenyan runners (Larsen, 2003; Larsen et al., 2004; Saltin, 2003; Saltin, Kim, et al., 1995; Saltin, Larsen, et al., 1995). Supposedly, it should require less energy to accelerate a lighter lower leg back and forward due to a lower segment moment of inertia. Scholz et al. (2008) measured foot length, lower leg length, lower leg volume, and lower leg moment of inertia in 15 Dutch well‐trained runners and found significant correlations between lower leg volume and RE and between lower leg moment of inertia and RE. However, after analysis for covariation with the Achilles tendon moment arm, they rejected the influence of these parameters (Scholz et al., 2008). In the present study, shank and foot volumes were measured by surface scanning, but no significant correlations were found between shank or foot volume and RE (Tables 5 and 6), although there were 56% difference between the smallest and the largest shank volume. Running experiments have shown that shod running is less expensive compared to barefooted but adding an extra weight of 100 g per shoe increased VO_2_ by 1% (Franz et al., 2012).

**TABLE 6:** Correlations (Spearman's Rho) between anthropometry and RE. * indicates a statistically significant correlation (Achilles tendon moment arm and RE at 18 km h^−1^). Foot ratio is Forefoot∙*L*_Ach_^−1^

	14 km∙h^−1^	18 km∙h^−1^
Leg length	Rho = 0.06 *p* = 0.85	Rho = 0.34 *p* = 0.28
Thigh length	Rho = 0.09 *p* = 0.77	Rho = 0.45 *p* = 0.14
Shank length	Rho = 0.18 *p* = 0.59	Rho = 0.46 *p* = 0.164
Foot length	Rho = 0.22 *p* = 0.49	Rho = 0.47 *p* = 0.12
Toe length	Rho = 0.08 *p* = 0.80	Rho = 0.37 *p* = 0.24
*L*_Ach_	Rho = 0.33 *p* = 0.30	Rho = 0.66 *p* = 0.02*
Shank vol.	Rho = 0.19 *p* = 0.56	Rho = 0.33 *p* = 0.30
Foot vol.	Rho = 0.27 *p* = 0.39	Rho = 0.49 *p* = 0.11
Foot ratio	Rho = −0.41 *p* = 0.18	Rho = −0.60 *p* = 0.043*

Different animal species exhibit often very different anatomy of the legs. Taylor et al. (1974) calculated that cheetahs, gazelles, and goats had equal energy cost moving their limbs during running despite large anatomical differences regarding limb mass, length, and distance to limb center of mass. It was therefore suggested that most of the energy expended in running at constant speed is not used to accelerate and decelerate limbs.

### 3.6 Fascicle length

In the present study, the fascicles of the soleus muscle showed a significant correlation to RE at 18 km h^−1^ when fascicle length was normalized to shank length (Figure 6). This indicated that longer muscle fibers have a positive influence on RE. However, this is not supported by the literature. On the contrary, Japanese runners were found to have longer GM fascicles than Kenyan runners (Sano et al., 2015). In the present study the medial gastrocnemius (GM) fascicles were 5.62 (0.72) cm on average (range: 4.50–6.84 cm), which corresponds very well the 5.36 (0.72) cm reported by Abe et al. (2000) for the GM in distance runners while 6.64 (1.32) cm for sprint runners. It was suggested that long muscle fibers would be beneficial for sprint runners due to a more optimal force–velocity relation (Abe et al., 2000; Lee & Piazza, 2009). Longer fascicles than in controls have also been reported for sumo wrestlers, and it was suggested that fascicle length may increase with strength training (Kearns et al., 2000).

Long fascicles imply long muscle fibers and more sarcomeres in series. As longer muscle fibers can contract at a higher shortening velocity than shorter fibers this would indicate a more beneficial force–velocity relationship of the muscles in question (Abe et al., 2001). At submaximal muscle activation, this means that the muscle can generate more force at the same shortening velocity. However, it is an important question whether the muscle fibers of, for example, the GM actually lengthen and shorten during running or the ankle joint movements are accomplished only by elastic length changes of the Achilles tendon. Giannakou et al. have shown that the GM fascicles stretch and shorten approximately 2.5 cm during running (11 km h^−1^) in 12 long‐distance runners (Giannakou et al., 2011) and Lai et al. found that the soleus fascicles covered 20% of the lengthening/shortening of the muscle–tendon unit during running at various speed (Lai et al., 2015). Since muscle strength is not related to the length of the muscle fibers, it is certain that longer muscle fibers with more sarcomeres in series consume more energy than shorter fibers when producing the same force (Walmsley & Proske, 1981). The force–length relationship of the muscle fibers is, on the other hand, highly influenced by fiber length, as longer fibers exhibit a wider range of length for optimal force production (Walmsley & Proske, 1981).

Finally, the most important feature of longer muscle fibers may be an altering of the force–velocity relation so that the muscle can produce more force at the same shortening velocity (Abe et al., 2000; Lee & Piazza, 2009). Only the relative length of the soleus fascicles correlated significantly to RE at 18 km h^−1^ in the present study (Figure 4), so additional research is required to establish whether long muscle fibers are an advantage regarding RE. This is especially interesting as reports exist showing that the number of sarcomeres in series can be increased in rats after downhill running (Lynn & Morgan, 1994; Lynn et al., 1998).

### 3.7 Stiffness and storage of elastic energy

A significant correlation between muscle–tendon stiffness and RE was found by Barnes et al. (2014). It was, however, poorly described how, exactly, stiffness was measured, but it was a maximal stiffness measured during vertical jumping. Barnes et al. also found a significant correlation between *L*_Ach_ and stiffness (Barnes et al., 2014), which was also found in the present study (Figure 7) where stiffness was measured during running. In both cases it seems that a short moment arm and a high stiffness follow each other and are somehow beneficial for RE. In Scholz et al. it was argued that a short moment arm of the Achilles tendon would imply a higher muscle force when producing a certain moment about the ankle joint as compared to a longer moment arm (Scholz et al., 2008). The higher muscle force would cause an increased stretch of the Achilles tendon during the eccentric contraction and thereby store more elastic energy in the tendon to be reused during the immediately following concentric contraction. This may certainly be true, but it requires the length and the stiffness of the tendon to match the muscle force exactly, so that the required range of joint motion is achieved.

Sano et al. found longer Achilles tendons in Kenyan than Japanese runners but also longer shanks. The cross‐sectional area of the Achilles tendon was also significantly larger than that of the Japanese (Kunimasa et al., 2014; Sano et al., 2015). Interestingly, given an upper limit on allowable tissue stress, a longer tendon would allow for storage of more elastic energy as would a stiffer tendon, which is the implication of a larger cross‐sectional area.

Another effect of a short Achilles tendon moment arm may be a positive influence on the force–velocity relation of skeletal muscles. At a given angular motion in the ankle joint during plantar flexion, a shorter *L*_Ach_ will cause a lower shortening velocity than a longer *L*_Ach_, simply due to geometry. In this way the required muscle force may be produced by fewer motor units and thereby fewer muscle fibers.

## 4 CONCLUSIONS

As the first study, we were able to show a significant correlation between biomechanical calculations of mechanical power and absolute oxygen consumption. However, this correlation did not exist when data were normalized to body mass. This is probably partly due to differences in anthropometry not accounted for in biomechanical movement analysis.

The Achilles tendon moment arm is considered highly important for RE as a short moment arm theoretically can be beneficial for both storage of elastic energy and for the force–velocity relation of skeletal muscles. The ratio between forefoot and Achilles tendon moment arm is also significantly correlated with RE due to a beneficial gearing of the foot with respect to the external forces. Stiffness of the whole body and the stance leg is indirectly important for RE as stiffness and Achilles tendon moment arm are significantly correlated. High stiffness of the leg muscles is very likely to favor storage and reuse of elastic energy during running.

## CONFLICT OF INTEREST

The authors declare no conflict of interest.

## AUTHOR CONTRIBUTION

C. E. Hansen planned and conducted the experiments, participated in the calculations, discussion of results, and in writing the manuscript. E. B. Simonsen planned and conducted the experiments, participated in the calculations, discussion of results, and in writing the manuscript. M. Stensvig participated in the biomechanical data collection and in writing the manuscript. J. Rasmussen participated in the biomechanical calculations, discussion of results, and in writing the manuscript. J. Wienecke participated in data collection and in writing the manuscript. J. Lorentzen participated in collection of data from ultrasonography, data interpretation, and in writing the manuscript. C. Villa participated in collection and interpretation of surface scans and in writing the manuscript.
